# New Dawn in the Treatment of Rheumatoid Arthritis: Advanced Insight into Polymer Hydrogel Research

**DOI:** 10.3390/gels11020136

**Published:** 2025-02-15

**Authors:** Shuai Wang, Jinyang Li, Fazhan Ren, Jiale Zhang, Wei Song, Lili Ren

**Affiliations:** 1 Key Laboratory of Bionic Engineering (Ministry of Education), College of Biological and Agricultural Engineering, Jilin University, Changchun 130022, China; swang23@mails.jlu.edu.cn; 2 College of Biological and Agricultural Engineering, Jilin University, Changchun 130012, China; 18514316688@163.com; 3 School of Agricultural Engineering and Food Science, Shandong University of Technology, Zibo 255000, China; renfazhan020113@163.com; 4 College of Engineering, Northeast Agricultural University, Harbin 150030, China; zhangjiale19811@163.com; 5 College of Engineering and Technology, Jilin Agricultural University, Changchun 130118, China; songwei@jlau.edu.cn; 6 National Key Laboratory of Automotive Chassis Integration and Bionics, Jilin University, Changchun 130022, China

**Keywords:** rheumatoid arthritis, pathophysiology, hydrogel, administration route, functionality

## Abstract

As a chronic systemic autoimmune disease, rheumatoid arthritis (RA) not only damages joints and other organs or systems throughout the body but also torments patients’ physical and mental health for a long time, seriously affecting their quality of life. According to incomplete statistics at present, the global prevalence of RA is approximately 0.5–1%, and the number of patients is increasing year by year. Currently, drug therapies are usually adopted for the treatment of RA, such as non-steroidal anti-inflammatory drugs (NSAIDs), disease-modifying antirheumatic drugs (DMARDs), glucocorticoids/steroids, and so on. However, traditional drug therapy has problems such as long half-lives, long treatment cycles requiring frequent drug administration, lack of specificity, and other possible adverse reactions (such as gastrointestinal side effects, skin stratum corneum barrier damage, and systemic toxicity), which greatly restrict the treatment of RA. In order to improve the limitations of traditional drug, physical, and surgical treatments for RA, a large number of related studies on the treatment of RA have been carried out. Among them, hydrogels have been widely used in the research on the treatment of RA due to their excellent biocompatibility, mechanical properties, and general adaptability. For example, hydrogels can be injected into the synovial cavity of joints as synovial fluid to reduce wear between joints, lubricate joints, and avoid synovial surface degradation. This article reviews the applications of hydrogels in the treatment of RA under different functions and the situation of hydrogels as carriers in the treatment of RA through different drug delivery routes and confirms the outstanding potential of hydrogels as drug carriers in the treatment of RA, which has great research significance.

## 1. Introduction

Rheumatoid arthritis (RA), as a chronic, systemic autoimmune disease with erosive, symmetrical polyarthritis as the main clinical manifestation, can not only damage joints, leading to joint pain, swelling, deformity, functional impairment, and damage to multiple organs or systems in the body, but also even cause premature death, seriously affecting the quality of life of residents [1,2,3]. It has been documented since 4500 BC and is caused by factors such as obesity, genetic mutations, injuries, autoimmune diseases, muscle weakness, and age-related joint wear [4]. Currently, the global prevalence of RA is approximately 0.5–1%. Moreover, according to the survey statistics, the incidence rate among women is significantly higher than that among men and the elderly [5]. There are multiple explanations for the pathogenesis of RA, such as genes, environmental factors, and abnormal immune responses [6]. Through autoimmune abnormalities, some cytokines are released, such as interleukins, leukotrienes, and other inflammatory factors (tumor necrosis factor) that may cause abnormal immune systems, thus resulting in the destruction of the body’s own physiological structures [7]. The pathological process of RA is very complicated and lengthy, and synovitis is the most fundamental pathological change in RA. As the disease progresses continuously, new blood vessels will gradually form inside the proliferating synovial tissue. These blood vessels, together with inflammatory cells, fibrous tissues, etc., will jointly form pannus. Once pannus is formed, it will further damage the articular cartilage and bone tissue and also interfere with the normal nutritional supply channels of the joints, causing the articular cartilage to gradually degenerate and wear [8]. Under the dual effects of long term inflammation and continuous erosion by pannus, the articular cartilage, and bone tissue will suffer extremely severe damage, leading to a narrowing of the joint space and eventually resulting in joint deformities [9]. Currently, the treatments for RA usually include physical therapy (such as thermotherapy and exercise therapy), drug therapy (non-steroidal anti-inflammatory drugs, antirheumatic drugs, glucocorticoids, etc.), and surgical treatment [10,11]. Although the existing treatment regimens can indeed play a positive role in patients with RA in practical applications, such as reducing the inflammatory response in patients, better controlling pain symptoms, and to some extent preventing the continuous development of joint damage. However, most of these methods have certain drawbacks and side effects, making it impossible to completely cure RA [12]. In order to treat RA, patients spend a lot of material and financial resources, which not only aggravate the physical and mental health of patients but also bring a heavy economic burden to them. Therefore, designing and developing new methods or new materials has become a clear goal and trend in the treatment of RA. Against this background, hydrogels have been gradually applied to the treatment research of RA due to their excellent biocompatibility, universal adaptability, degradability, controllability, and other properties [13]. Unlike previous reviews on hydrogel treatment of RA, which focused on hydrogel preparation materials and stimulus responsiveness, this article focuses on the functions and administration methods of hydrogels during RA treatment. By integrating the latest research on hydrogel-based RA therapies, it comprehensively summarizes the pathogenesis and treatment plans of RA. It not only covers the excellent characteristics of hydrogels but also reviews the practical applications of hydrogels with different functions in treating RA. Moreover, it deeply explores the use of hydrogels as drug carriers for RA treatment via various administration routes, validating the potential of hydrogels in treating RA. This offers new references and insights for future RA treatment with hydrogels, thus being of great research significance.

## 2. Pathophysiology of RA

**Figure 1:**
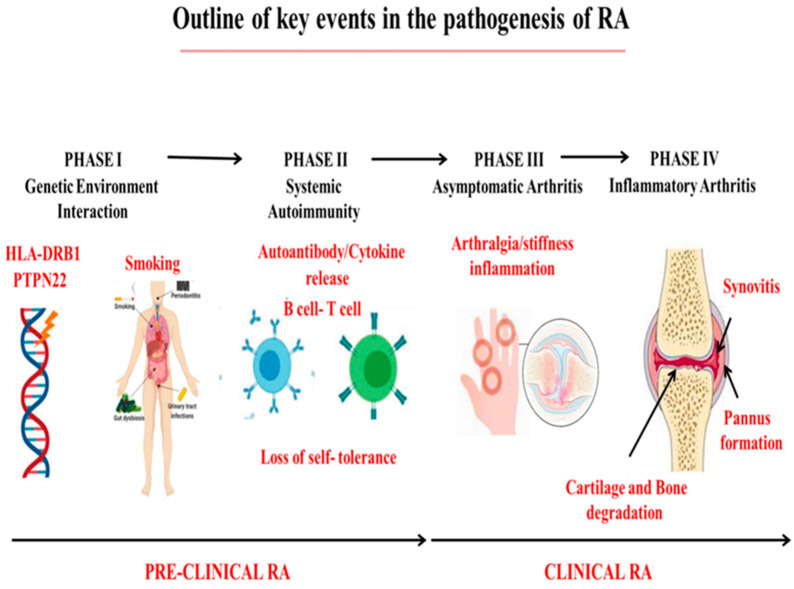
Outline of key events in RA pathogenesis: Genetic and environmental factors play a role in triggering autoimmunity, and preclinical RA is characterized by the presence of irregular biomarkers of inflammation [16]. Copyright © 2023 Elsevier B.V.

The pathogenesis of RA is rather complicated. Currently, there are three main scientific explanations for its pathogenesis, which mainly include genetic factors, abnormal activation of immune cells, and induction by environmental factors. Figure 1 systematically summarizes the pathogenesis of RA joints and the related inflammatory mechanisms compared with normal joints.

### 2.1. Genetic Factors

Multiple studies have reported that RA has a certain genetic predisposition. It is estimated that genetic factors account for approximately 50–60% of cases of RA among patients. The disease-causing genes of RA are likely to be inherited by offspring through mitosis and meiosis. Currently, based on relevant studies of the whole genome, single nucleotide polymorphisms (SNPs) among more than 150 susceptibility genetic factors have been identified as contributing factors to the induction of RA disease [14]. In addition, the human leukocyte antigen (*HLA*) *HLA-DRB* gene is closely related to the pathogenesis of RA [15]. *HLA* is the gene product of the major histocompatibility complex located on the short arm of human chromosome 6. The protein molecules encoded by *HLA* play a crucial role in the process of immune response and immune recognition. *HLA* includes multiple types of molecules, such as class I and class II molecules. The *HLA* class I molecule is composed of a heavy chain and a light chain and is expressed on the surface of almost all nucleated cells [16]. Its core function is to present endogenous antigen peptides, such as viral protein fragments synthesized inside infected cells, to cytotoxic T lymphocytes (CTL). In this way, the cellular immune response mechanism can be initiated, enabling CTL to accurately identify and kill the self-cells infected by viruses and other pathogens. The *HLA* class II molecules are mainly expressed on the surface of antigen-presenting cells (such as dendritic cells, macrophages, and B cells). It consists of an α chain and a β chain, and its main function is to present exogenous antigen peptides to helper T lymphocytes, activate Th cells, and then trigger humoral and cellular immune responses [17]. The formation of *HLA* class II antigens is encoded by DR, DP, and DQ loci. Among different alleles of *HLA*, many studies have confirmed the involvement of *HLA-DRB1* and the correlation between this gene and the pathogenesis of RA is up to 30% [18]. In RA genetics, specific alleles of the *HLA-DRB1* gene are a key factor. These alleles are inherited in a codominant manner, that is, the alleles from both parents will be expressed, and inheriting these RA-related alleles increases the risk of children developing RA. Furthermore, as a polygenic disease, in addition to the *HLA* genes, there are also other non-human leukocyte antigen genes that can induce the onset of RA [19]. These related genes are distributed in different segments at different positions on chromosomes and increase the prevalence of RA through their interactions. For example, when the *PTPN22* gene (protein tyrosine phosphatase non-receptor type 22 gene), which plays a role in regulating the signal transduction process of T cells and B cells, mutates, it may lead to easier activation of immune cells, thereby increasing the risk of RA [20]. Moreover, different disease-causing genes interact with each other. For example, when the *HLA* gene interacts with the *PTPN22* gene, the risk of developing RA is higher than that of individuals carrying only one of the gene mutations. This interaction may be achieved by affecting autoimmunity. For example, the *HLA* gene can affect the antigen presentation process, increasing the probability that self-antigens are recognized by the immune system. Meanwhile, after the mutation of the *PTPN22* gene, immune cells will overrespond to the presented antigens. The combined action of the two further increases the possibility of RA onset [21].

### 2.2. Abnormal Activation of Immune Cells

**Figure 2:**
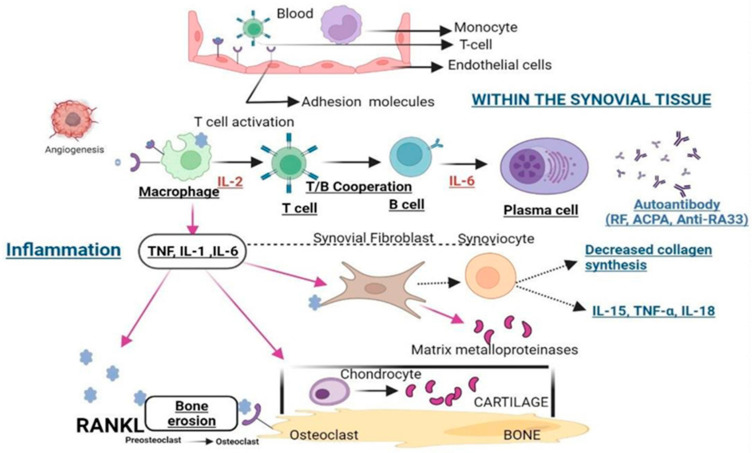
Pathophysiology of RA. Abbreviations: TNF-α: tumor necrosis factor-alpha; IL-1: interleukin-1; IL-2: interleukin-2; IL-6: interleukin-6; IL-15: interleukin-15; IL-18: interleukin-18; RF: rheumatoid factor; ACPA: anti-citrullinated peptide antibody; RANKL: Receptor activator of nuclear factor kappa-B ligand [23]. Copyright © 2023 Elsevier B.V.

In addition, the abnormal activation of immune cells is also a key factor in inducing RA [22]. As an autoimmune disease, the autoimmune response of RA involves multiple immune cells, such as T cells, B cells, and macrophages. Figure 2 shows the main immune cells and substances involved in the pathogenesis of RA.

Under a normal immune system, the activation of T cells is a highly precise and strictly regulated process. Antigen-presenting cells (APC) need to take up and process foreign or self-antigens and then present the antigen peptide-molecule complexes to the T cell receptor. Meanwhile, with the synergistic effect of co-stimulatory molecules and so on, T cells will be moderately activated [24]. However, when RA occurs, the originally orderly activation process will fall into a chaotic state. For example, some self-antigens may be affected and show abnormal conditions, or environmental factors may induce changes in the antigen structure, etc., which then leads to the incorrect presentation of these self-antigens by APC to T cells. Especially in individuals carrying specific *HLA* genotypes, the *HLA* molecules on their APC are more likely to bind to self-antigen peptides and then present these self-antigen peptides to T cells, thus breaking immune tolerance. The activated T cells will proliferate in large numbers and secrete multiple cytokines, such as interleukins and interferons [25,26]. These can further promote the proliferation and differentiation of T cells, forming more effector T cells. In addition, these cytokines can further activate several cells involved in the immune response and enhance their inflammatory activities. Moreover, these T cells will migrate to the synovial tissue of joints and interact with synovial cells, constantly attracting more immune cells to enter the local area of joints, making the inflammatory response in the synovial tissue of joints persist and continuously intensify, unable to terminate normally, laying the foundation for the occurrence and development of RA [27].

B cells, as crucial lymphocytes, also play a key role in the occurrence and development of RA [28]. Mature B cells are activated under the interaction with T cells when they act as antigen-presenting cells themselves or are promoted by the cytokines secreted by T cells. Then, B cells will differentiate into plasma cells. These plasma B cells produce and secrete a large number of autoantibodies, such as rheumatoid factor (RF) and anti-citrullinated protein antibodies (ACPA) [29,30]. Rheumatoid factor (RF) is a kind of autoantibody, and its target is the Fc segment of IgG. RF can combine with the body’s own IgG to form immune complexes. When these immune complexes are deposited in the synovial tissue of joints and other parts, they will activate the immune system and prompt the production of certain anaphylatoxins. These anaphylatoxins can attract inflammatory cells such as neutrophils to gather at the joint sites. Subsequently, the inflammatory cells will release inflammatory mediators, causing damage to the joint tissues [31]. ACPAs are highly specific autoantibodies for RA. They have the ability to specifically recognize citrullinated proteins and can destroy the structures related to joints through immune reactions, playing a key role in the pathogenesis of RA. In addition, B cells can not only produce various autoantibodies but also have the function of antigen-presenting cells themselves. They can present the antigens they have taken up to T cells again. This further strengthens the activation of T cells and continuously amplifies the entire immune response. More and more immune cells will be activated, and more inflammatory mediators will be released, ultimately leading to the increasing severity of the inflammation in the synovial tissue of joints and gradually promoting the onset of RA [32].

Furthermore, studies have confirmed that macrophages play an important role in persistent synovial inflammation and arthritis damage [33,34]. In the context of RA, macrophages can be abnormally activated by multiple factors. Macrophages are usually divided into pro-inflammatory M1-like and anti-inflammatory M2-like phenotypes. M1-like macrophages continuously secrete inflammatory cytokines. Among them, tumor necrosis factor-α (TNF-α), interleukin-1 (IL-1), and interleukin-6 (IL-6) play key roles in triggering RA [35,36]. TNF-α has the ability to induce synovial cells and chondrocytes to produce matrix metalloproteinases (MMPs). The production of MMPs will degrade the extracellular matrix of articular cartilage and bone tissue, thereby causing damage to the joint structure [37]. IL-1 can, on the one hand, stimulate the proliferation of synovial cells, making the synovial tissue continuously proliferate and thicken; on the other hand, it can also enhance the activities of other inflammatory cells (such as neutrophils and lymphocytes, etc.), promoting the continuous intensification of the inflammatory response and making it difficult to subside [38]. IL-6 plays a role in the process of B cell differentiation into plasma cells and antibody production, promoting the generation of more autoantibodies and further aggravating immune disorders and joint damage [39].

Therefore, as an autoimmune disease, RA is closely related to the abnormal functions of various immune cells in the immune system.

### 2.3. Environmental Factors

The factors that trigger RA are related not only to internal factors of the body itself but also to external factors. Currently, numerous studies have shown that environmental factors can also induce RA. Although environmental factors have a limited role in the pathogenesis of RA, a series of studies have emphasized their significant impact on the development of the disease. The dietary habits, dust, obesity, gut microbiota, periodontitis, and viral infections (Parvovirus B19, Cytomegalovirus, Epstein-Barr virus, Hepatitis B and C viruses, and Herpes simplex virus) that have been reported so far are also involved in the pathogenesis of RA and may all lead to the occurrence of RA [40]. For example, after the human body is infected with the Epstein-Barr virus, the viral proteins may mimic self-antigens, triggering a cross-reaction and activating the immune system [41]. Moreover, among the environmental factors studied in the pathogenesis of RA, smoking is a definite environmental risk factor and can greatly increase the incidence rate of RA [42,43]. This is mainly because the chemicals in cigarettes can citrullinate proteins, resulting in the production of ACPA and increasing the risk of developing RA. Meanwhile, smoking can also affect the function of the immune system, making inflammatory reactions more likely to occur.

In summary, the pathogenesis of RA is rather complicated. It is related not only to genetic factors and the abnormal activation of immune cells but also to environmental factors, which makes the treatment of RA even more complicated. Therefore, it is an indispensable step to seek a good treatment method for RA.

## 3. Overview of Methodologies for RA Treatment

As can be seen from the above pathogenesis of RA, the pathogenesis of RA is so complicated that its treatment becomes increasingly complex. There is currently no way to completely treat RA. The treatments can only relieve pain, eliminate inflammation, suppress the abnormal function of the immune system to reduce the damage to human bones and cartilage, and maintain joint functions as much as possible. At present, the main treatment methods for RA mainly include physical therapy, drug therapy, and surgical treatment.

### 3.1. Physical Therapy

Physical therapy mainly includes thermotherapy, cryotherapy, and massage. Thermotherapy promotes local blood circulation of the skin through thermal stimulation (such as hot spring baths, hot compresses, and hot baths), helps muscles relax, relieves muscle spasms, and alleviates pain and stiffness of the limbs. Cryotherapy means that when a joint inflammation has an acute onset and local redness, swelling, heat, and pain appear, appropriate cold compress can constrict blood vessels, reduce inflammatory congestion and swelling, and play a role in relieving pain [44]. In addition, moderate massage can also relax muscles and relieve joint pain to some extent and improve the joint’s motor function [45]. However, physical therapy has relatively limited effects on the treatment of RA and can only play an auxiliary role rather than a key role.

### 3.2. Drug Therapy

Drug therapy is currently the main approach for treating RA. Drugs can be classified into non-steroidal anti-inflammatory drugs (NSAIDs), glucocorticoids/steroids, disease-modifying antirheumatic drugs (DMARDs), biologics, traditional Chinese medicine, etc. [46]. NSAIDs mainly include aspirin, ibuprofen, naproxen, diclofenac, celecoxib, rofecoxib, piroxicam, along with others. NSAIDs mainly work by inhibiting the activity of cyclooxygenase to prevent the production of inflammatory factors (such as prostaglandins, prostacyclin, and thromboxanes), which can effectively relieve pain, joint inflammation, redness, and swelling. However, long term use may lead to adverse gastrointestinal reactions, such as ulcers, abdominal pain, and bleeding, and may also affect liver and kidney functions [47,48]. Glucocorticoids/steroids mainly include prednisolone, prednisone, dexamethasone, betamethasone, and so on [49]. Glucocorticoids/steroids inhibit the activity of eosinophils by preventing the release of phospholipids, thus rapidly relieving the inflammation and pain caused by RA. However, long term use of such drugs will reduce the production and release of the body’s own natural steroids and have a negative impact on protein and fat metabolism in the body, triggering diseases such as decreased bone density and diabetes. DMARDs have been widely used since 1980 due to their antirheumatic effects. Conventional synthetic DMARDs mainly include methotrexate (MTX), sulfasalazine, leflunomide, hydroxychloroquine, azathioprine, cyclosporin, cyclophosphamide, gold salts, and penicillamine [50,51]. These drugs can improve and delay the progress of the disease, fundamentally regulate the immune function of the body, and inhibit pathological processes such as abnormal activation of immune cells. However, this group of drugs will not relieve pain and needs to be used in combination with other therapeutic drugs to improve efficacy, reduce the dose of a single drug, and reduce the risk of adverse reactions. Biologics mainly include TNF-α antagonists, interleukin-6 (IL-6) antagonists, B cell depleting agents, and so on [52]. Through targeted therapy aiming at specific cytokines or cell surface molecules involved in the pathogenesis of RA, the inflammatory pathways are specifically blocked, thereby achieving the purpose of controlling the disease. Although biological agents have remarkable curative effects, there are problems such as the risk of infection (such as tuberculosis infection, fungal infection, etc.), injection site reactions, and inducing autoimmune diseases.

### 3.3. Surgical Treatment

In addition, apart from the above treatment methods, surgical treatment can serve as the final treatment option for RA. Surgical treatment mainly includes synovectomy, artificial joint replacement, and arthrodesis, etc. Synovectomy aims to reduce the production of inflammatory factors, delay the destruction of articular cartilage and bone, improve joint function to some extent, and relieve pain by removing the diseased synovial tissue. For artificial joint replacement, when RA causes severe damage to the joints, resulting in joint deformities and serious loss of function, and when patients’ basic life functions, such as walking and standing, are greatly affected, artificial joint replacement can be considered. When it comes to arthrodesis, it is adopted when the condition is severe [53]. Arthrodesis fixes the diseased joint in a functional position to eliminate joint pain, allowing patients to carry out some basic limb movements in a relatively stable and pain-free state. Although surgical treatment can play a role in the treatment of RA, risks such as postoperative recurrence and wound infection cannot be avoided. Moreover, drugs are still needed after the operation, which may aggravate the patient’s condition and bring about related adverse reactions.

Therefore, it is very difficult to find a method that can both have a good effect on treating RA and cause fewer side effects. For this reason, it is of great importance to seek a good treatment method for RA.

## 4. Advantages of Hydrogels in the Treatment of RA

Given the numerous drawbacks of the current treatment methods for RA, a substantial number of studies have been conducted to develop new biomaterials and medical drugs as well as treatment approaches for RA. Among these, hydrogels, thanks to their outstanding properties, have been employed in medical research aiming at treating RA.

### 4.1. Biocompatibility and Antibacterial Properties

The raw materials for currently prepared medical hydrogels are usually natural materials and synthetic materials. Natural materials include chitosan, cellulose, hyaluronic acid, gelatin, sodium alginate, silk fibroin, collagen, chondroitin sulfate, and so on [54,55]. Synthetic materials include polyvinyl alcohol (PVA), polyethylene glycol (PEG), and others [56,57]. These natural/synthetic materials possess excellent biocompatibility and antibacterial properties, which are crucial for the treatment of RA. Biocompatibility and antibacterial properties mean that when hydrogels are used for the treatment of RA, they will not cause rejection reactions with the tissues and cells around the joints and will not lead to wound infections. In the human body, foreign substances are prone to triggering immune rejection reactions. However, hydrogels with good biocompatibility can be accepted and tolerated by the body’s immune system can remain in the body for a long time and will not produce serious adverse reactions due to immune rejection, which is extremely important for diseases such as RA that require long term treatment [58]. In addition, joints affected by RA have conditions such as inflammation and tissue damage. Hydrogels with good biocompatibility can integrate well into the existing biological environment and can adapt to various physiological conditions of joint tissues, such as pH, ion concentration, and the presence of multiple enzymes and proteins. This adaptability helps hydrogels maintain stability and keep their functions normal and also facilitates them to play their expected functions, such as drug delivery and regulation of the inflammatory microenvironment. Therefore, the biocompatibility and antibacterial properties of hydrogels are indispensable for the treatment of various diseases, including RA.

### 4.2. Adjustable Physical Properties

Hydrogels, which are composed of hydrophilic polymers, can be cross-linked through various methods. Physical cross-linking approaches such as hydrogen bond cross-linking, ion cross-linking, and electrostatic effects can be employed, and chemical cross-linking can also be achieved via photo-cross-linking, enzyme cross-linking, and Schiff base cross-linking, or click chemical reaction [59]. By relying on these different cross-linking methods, the mechanical properties of hydrogels could be regulated to obtain the medical hydrogels that meet specific requirements. When it comes to the elastic modulus of hydrogels, it can be adjusted by altering the composition of the hydrogels as well as the cross-linking density. In the context of treating RA, an appropriate elastic modulus allows hydrogels to function as effective buffers within the joint cavity. They can mimic the mechanical characteristics of natural articular cartilage, efficiently dispersing stress during joint movements. This, in turn, helps to reduce the adverse impacts caused by friction and compression between bones and damaged tissues, safeguarding the joint structure from further deterioration [60]. For example, the Gelatin Methacrylate (GelMA)/Poly(2-ethyl-2-oxazoline) hydrogel, loaded with the drug Kartogenin, the GelMA demonstrates a high swelling capacity, swelling up to 8-10 times its dry weight. Its elastic modulus is 707.31 ± 0.11 kPa, closely approaching the range of natural articular cartilage (500–900 kPa). Moreover, research has proven that in the environment of damaged cartilage tissue, it also exhibits good blood compatibility and sustained drug release, which is beneficial to cartilage regeneration [61]. Toughness is equally crucial for hydrogels in RA treatment. High-toughness hydrogels can maintain their integrity and avoid deformation or failure even during frequent joint activities. Suitable polymer materials and an optimized preparation process were selected to adjust the toughness of hydrogels, ensuring that they remain intact within the complex mechanical environment of joints over an extended period and continue to perform their functions stably. Moreover, appropriate swelling degree and swelling rates enable hydrogels to conform better to the shape of the joint cavity and fill the joint gaps. Furthermore, the swelling process can be harnessed to achieve controlled release of drugs. The swelling degree of hydrogels can be adjusted as per requirements. For instance, the desired swelling state was obtained by adjusting the number of hydrophilic groups and the density of the cross-linking network. Different swelling rates can also be designed based on specific needs. For example, the traditional polyacrylamide/sodium alginate double-network structure, due to the loose structure of sodium alginate, usually fails to meet the required mechanical and anti-swelling property requirements. However, the polyacrylamide/sodium alginate double-network hydrogel prepared by a modified freeze-casting strategy combining freeze-casting and re-freezing can exhibit excellent anti-swelling performance (32.26%), high toughness (5.291 MJ/m^3^), and high tensile strength (3.491 MPa) [62]. Stiffness is also an indispensable physical property of hydrogels. Research shows that different hydrogel stiffnesses can affect the polarization of senescent macrophages (S-MΦs). For instance, polyacrylamide hydrogels with a stiffness of 18 kPa and 76 kPa induce the anti-inflammatory phenotype of S-MΦs, while polyacrylamide hydrogels with a stiffness of 295 kPa induce the pro-inflammatory phenotype of S-MΦs. This indicates that adjusting the stiffness of implant biomaterials can effectively influence the polarization of S-MΦs, which contributes to the regeneration and repair of aging bone tissue, reduces the occurrence of related inflammation, and is beneficial for the treatment of RA [63]. In addition, the degradability of hydrogels can be adjusted according to the disease condition. For example, once degradable hydrogels have fulfilled functions such as sustained drug release and physical protection, they will decompose naturally without the necessity for additional complicated procedures such as removal, thereby reducing the risk of harm to the human body. In summary, the adjustability of hydrogels facilitates the treatment of RA and is an essential factor in this regard [64].

### 4.3. Lubricating Properties

In human joints, the natural synovial fluid plays a vital lubricating role in facilitating the normal activities of joints. Components such as hyaluronic acid and lubricin within the synovial fluid can effectively mitigate the friction between joint surfaces. From the perspective of the pathogenesis of RA, it was known that when patients suffer from RA, the synovial fluid gradually decreases, which subsequently results in the gradual wear of joints and exacerbates the condition. Hence, joint lubrication is of certain significance in the treatment of RA and in alleviating pain. Hydrogels, being an elastic and porous material, have their polymer matrices encapsulating a substantial amount of water, therapeutic drugs, or other solutions. In terms of composition and function, hydrogels can mimic the synovial fluid. With a large quantity of liquid components and a hydrophilic polymer network, they possess lubricating capabilities similar to those of the synovial fluid. They can serve as temporary “artificial synovial fluid” in the joint cavity, thus ensuring the smooth operation of joint activities and relieving the issues of limited mobility and pain caused by factors such as joint inflammation and cartilage wear in RA patients [65]. Furthermore, hydrogels possess a certain degree of elasticity and softness. When placed in areas such as the joint cavity, they can function as a buffer against external pressure, reducing the friction and collision between bones and damaged tissues during joint movements and offering physical protection to the joints. Moreover, hydrogels can fill in the minute defects of articular cartilage form a lubricating layer at the defective areas, prevent friction at those spots, and slow down their further deterioration. Additionally, they can cover the surface of the synovial membrane, reduce the friction between the synovial membrane and other tissues, and relieve symptoms such as synovial membrane hyperplasia and pain induced by RA inflammation. Consequently, the lubricating properties of hydrogels are beneficial to the treatment of RA.

### 4.4. Structural Superiority

The unique reticular or porous structure of hydrogels endows them with the excellent property of being outstanding drug carriers, allowing them to encapsulate drugs relevant to the treatment of RA within themselves. As known from the account of drug therapies for RA, most drugs currently available must be taken in small doses and multiple times, which is not only a tiresome process but also prone to delaying the treatment. Thanks to their distinctive structure, hydrogels can enable drugs to be released slowly and continuously from the gels, thus realizing a long acting and controlled drug release effect. This helps reduce the inconvenience brought about by frequent drug administration, maintain a relatively stable drug concentration in the body, and more effectively allow the drugs to continuously exert their curative effects to keep the progression of RA in check. Moreover, hydrogels generally exhibit structural stability. In relevant in vivo experiments for RA treatment, hydrogels can exist relatively stably without being rapidly degraded. Furthermore, hydrogels can mimic the structure of the extracellular matrix and form a three-dimensional network structure. In the local area of joints, they can play a certain regulatory role in aspects such as the local cytokine environment and immune microenvironment. For instance, they can absorb or neutralize inflammatory cytokines and inhibit local abnormal immune responses, thereby improving the local inflammatory state of RA at its root and facilitating the repair and functional recovery of joint tissues [66]. Consequently, the excellent structure of hydrogels makes them the optimal choice for treating RA.

All in all, with their remarkable biocompatibility, antibacterial properties, adjustability, lubricating properties, as well as excellent structural features, hydrogels demonstrate great potential in the treatment of RA. Hence, the paper reviews the practical applications of functional hydrogels used for RA treatment in recent years and the application scenarios of hydrogels serving as carriers for the treatment of RA through different drug administration routes. It verifies the potential of hydrogels in treating RA and holds significant research value.

## 5. Functional Hydrogels for the Treatment of RA

Hydrogels have emerged as an important biomaterial, primarily due to their three-dimensional hydrophilic network structure and a host of other outstanding properties. These include excellent biocompatibility, antibacterial capabilities, high adjustability, and the advantage of having a wide variety of raw materials available for their preparation. Moreover, hydrogels can mimic the structure of the extracellular matrix. This enables them to create a favorable environment for cells, thereby facilitating cell proliferation and accelerating the repair of damaged areas [67]. As a result, hydrogels have been increasingly applied as biomedical materials in multiple aspects of the medical field, such as tissue repair, wound healing, and drug delivery. They are also being utilized to treat a range of related diseases. In recent years, a significant number of studies have demonstrated that hydrogels hold great potential in the treatment of RA. During the progression of RA, hydrogels can perform different functions in response to various symptoms. More specifically, hydrogels used for RA treatment can be categorized into several types according to the functions they display, namely tissue engineering scaffold hydrogels., lubricating hydrogels, immunomodulatory hydrogels, tissue repair hydrogels, and drug delivery hydrogels. Table 1 summarizes the functions exhibited by different types of hydrogels in the treatment of RA.

### 5.1. Tissue Engineering Scaffold Hydrogels

**Figure 3:**
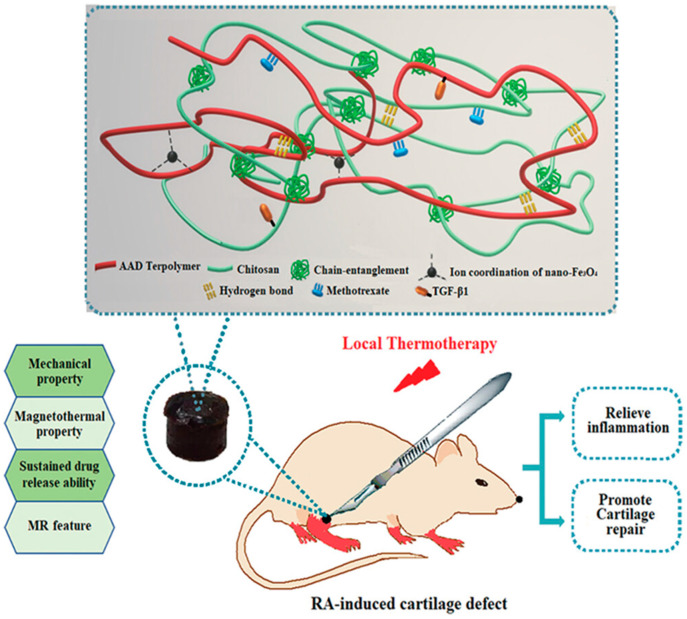
Schematic illustration of drug-loaded multifunctional hydrogels for relieving inflammation and promoting cartilage defects in RA [72]. Copyright © 2021 Wiley-VCH GmbH.

During the progression of RA, abnormalities within the immune system can lead to the gradual degeneration and wear of articular cartilage. As a result of prolonged erosion, both the articular cartilage and bone tissues endure severe damage, causing the joint space to narrow progressively. The absence of adequate support and cushioning then heightens the friction between bone joints, further worsening the condition of the disease. Tissue engineering scaffold hydrogels can be utilized to fill the joint cavity. Thanks to their suitable mechanical properties, these hydrogels are capable of mimicking certain functions of natural articular cartilage, thereby fulfilling the roles of cushioning and supporting. For instance, Fang et al. constructed a multifunctional hydrogel (DN-Fe-MTX-TGFβ1) by incorporating the antirheumatic drug methotrexate DN-Fe-MTX-TGFβ1 and transforming growth factor β1 (TGF-β1) into a nano-Fe3O4 composite chitosan-polyolefin. Figure 3 is a schematic diagram of the drug-loaded multifunctional hydrogel. This hydrogel boasts excellent mechanical properties, enabling it to serve as a reliable supporting scaffold to ensure structural stability, just like articular cartilage does. Moreover, it has the capacity for continuous drug release and exhibits magnetothermal properties. Acting as a carrier, it allows for the slow release of MTX and TGF-β1. During the 2-month monitoring of hydrogels for sustained-release drugs in vitro, both MTX and TGF-β1 were released slowly without any bursts in release rate, thereby minimizing the toxicity caused by the accumulation of drugs in the target tissues. Additionally, due to its magnetothermal effect, the hydrogel can continuously supply local thermochemistry for RA. In vivo experiments on rats have demonstrated that this hydrogel can promote cartilage regeneration, which opens up possibilities for the combined development of tumor-inspired therapies and tissue engineering [72].

Furthermore, some hydrogels possess the functions of stimulating the proliferation and differentiation of local cells and accelerating the repair of damaged tissues. For example, Zhao et al. designed a nanoenzyme-enhanced hydrogel. By taking advantage of the endogenous hydrogen peroxide (H_2_O_2_) present in the joints of RA and encapsulating enzymes with similar catalase activity to catalyze its decomposition, this hydrogel can break down H_2_O_2_ into water and oxygen. Consequently, it increases the local oxygen content in the joints, creating a favorable environment for the survival and proliferation of stem cells. This, in turn, safeguards the implanted cells from death and osteogenesis limitations caused by reactive oxygen species and hypoxia, ultimately enhancing the efficacy of stem cell therapy for RA [73].

### 5.2. Lubricating Hydrogels

**Figure 4:**
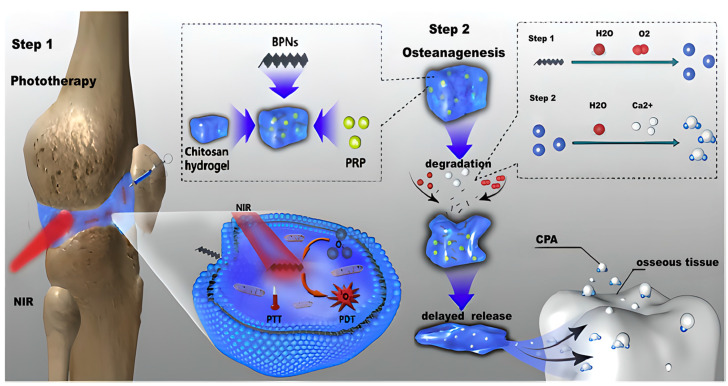
Schematic illustration of the PRP-Chitosan thermoresponsive hydrogel combined with black phosphorus nanosheets as injectable biomaterial for biotherapy and phototherapy treatment of RA [76]. Copyright © 2023 Elsevier B.V.

During the course of RA, the articular cartilage will gradually degenerate and wear away. This also affects the normal secretion and function of joint synovial fluid, resulting in insufficient joint lubrication. Under the influence of long term inflammation, the articular cartilage and bone tissues will suffer extremely severe damage, leading to increased joint friction and aggravated pain. Lubricating hydrogels can form a protective film within the joints, isolating the diseased synovial tissues, cartilage, and other components from possible external stimulating factors. Moreover, they can simulate the lubricating properties of natural synovial fluid, fill the spaces between the surfaces of articular cartilage, effectively reduce the friction during joint activities, and relieve the pain and cartilage wear caused by friction [74]. Hu et al. prepared a novel polyvinyl alcohol/polyethylene glycol/graphene oxide (PVA/PEG/GO) hydrogel by physical cross-linking. Due to the formation of a strong hydrogen bond network between the polymer chains and GO, and the high content of a large number of hydrophilic groups, such as carboxyl and hydroxyl groups on the surface, it can absorb and retain a large amount of water, demonstrating excellent lubricating properties. In addition, the PVA/PEG/GO hydrogel has a superior self-healing function and does not break under a weight of 200 g. Because of its excellent sustained-release lubricating properties and self-healing properties, it provides a new candidate material for the treatment of RA [75]. Pan et al. combined platelet-rich plasma (PRP)/chitosan thermosensitive hydrogel with black phosphorus nanosheets (BPNs) to fabricate an injectable biomaterial for the treatment of RA. Figure 4 shows the design strategy of the hydrogel system. When exposed to near-infrared light irradiation, BPNs generate heat and deliver reactive oxygen species (ROS) to the inflamed joints to eliminate the proliferative synovial tissues. The degradation products of BPNs can be controlled and released by the hydrogel; experiments show that in the first 48 h, degradation speed declined fastest and then kept stable, providing essential raw materials for bone regeneration. Moreover, the hydrogel’s lubricating properties enable it to reduce the friction on the surrounding tissues, thereby safeguarding the articular cartilage. This work offers valuable insights into the application of thermoresponsive hydrogels in the treatment of RA [76].

### 5.3. Immunomodulatory Hydrogels

Since RA is an autoimmune disease, immunomodulation plays a crucial role in its treatment. Immunomodulatory hydrogels can regulate the local immune microenvironment in joints by adsorbing or neutralizing inflammatory cytokines, interfering with the activation and proliferation of immune cells, and so on, thereby alleviating the symptoms of RA [77]. For example, Liao et al. designed a self-assembling Nap-DFDFDEGPIRRSDS (abbreviated as FP) peptide and incorporated it into an injectable supramolecular hydrogel, which could inhibit TNF-α within 15 min. Moreover, after integrating metformin-loaded hollow copper sulfide (CuS/MET, abbreviated as CM) nanoparticles (NPs) into the hydrogel, the hydrogel could reduce the polarization of RA synovial fibroblasts and pro-inflammatory M1 macrophages by inhibiting the production of TNF-α and releasing metformin. In addition, copper sulfide nanoparticles have the effect of promoting articular cartilage formation. It has been verified in mouse experiments that this hydrogel can significantly relieve synovial inflammation and promote cartilage repair. This method holds promise for the treatment of various inflammatory arthritides with overexpressed TNF-α, such as RA, ankylosing spondylitis, and psoriatic arthritis [78].

**Figure 5:**
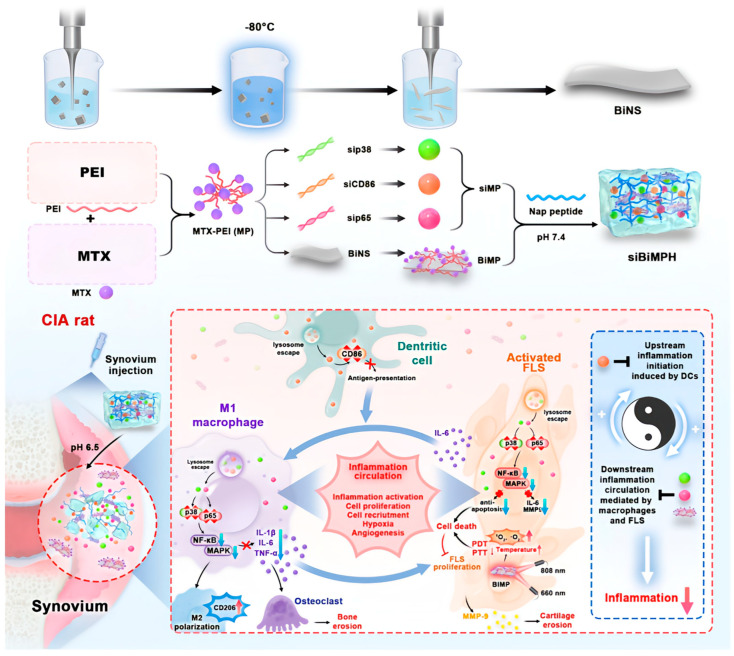
Schematic illustration of the PRP-Chitosan thermoresponsive hydrogel combined with black phosphorus nanosheets as injectable biomaterial for biotherapy and phototherapy treatment of RA. PEI: polyethyleneimine, MTX: methotrexate, BINS: bismuthene nanosheets, siRNA: a double-stranded RNA, TNF-α: tumor necrosis factor-α, IL-1: interleukin-1, IL-6: interleukin-6, siBiMPNH: pH-responsive injectable peptide hydrogel [79]. Copyright © 2023 Elsevier B.V.

Wu et al. developed a pH-responsive injectable peptide hydrogel (siBiMPNH) that encapsulated a new two-dimensional nanosheets and gene transfection complexes, including sip65MP, sip38MP, and siCD86MP. Two-dimensional (2D) nanosheets, as a new type of ultrathin multi-layer (single-atom or multi-atom) materials with high anisotropy and planar structures, have been widely used in cancer treatment. Among bismuth-based materials, 2D bismuth nanosheets possess a special structure. One of their most important characteristics is the smallest longitudinal dimension, which endows them with high reactivity and excellent photothermal conversion efficiency. Meanwhile, the planar nano-heterostructure significantly promotes the photo-induced electron-hole separation, thus enhancing the generation of ROS, which is beneficial for photodynamic therapy. Figure 5 shows the preparation and treatment strategies of siBiMPNH. Due to the excessive proliferation of fibroblasts and the excessive aggregation of immune cells during the course of RA, the inflamed synovial microenvironment shows acidity. Therefore, after the hydrogel is injected into the synovium, siBiMPNH will quickly respond at a lower pH value, causing the hydrogel to collapse and then accelerating the release of nanoparticles. For example, when the concentration of Nap peptide is 25 mg/mL, the cumulative release percentage of siMP within 48 h is 49.89% at pH 6.5 and drops to 38.37% at pH 7.4. The cumulative release percentages within 7 days are 93.57% and 70.40% at pH 6.5 and pH 7.4, respectively. The cumulative release rates of BiMP within 7 days at pH 6.5 and 7.4 are 83.47% and 63.21%, respectively. Among them, siCD86MP directly inhibits the expression of the inflammatory factor CD86. Sip65MP and sip38MP inhibit the nuclear factor-κB and mitogen-activated protein kinase pathways of macrophages and synovial fibroblasts, thereby reducing the secretion of TNF-α, interleukin-6 (IL-6), interleukin-1β (IL-1β), and matrix metalloproteinase 9, achieving the therapeutic effect on RA [79].

### 5.4. Drug Delivery Hydrogels

**Figure 6:**
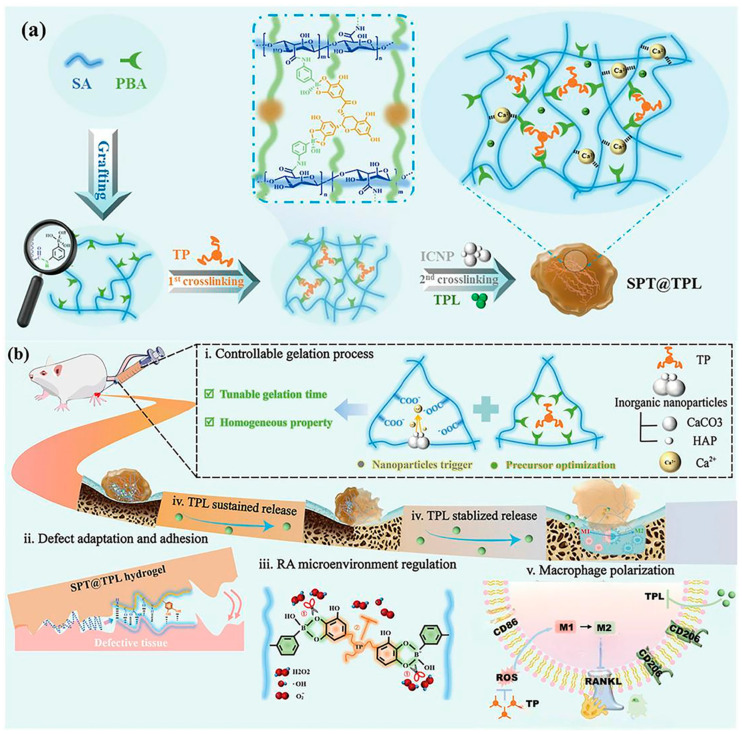
Synthesis (**a**) and function (**b**) schematic of SPT@TPL hydrogel for RA treatment. SA: Sodium alginate, TPL: triptolide, PBA: 3-Aminophenylboronic acid, TP: Tea polyphenols, SPT@TPL: dual dynamically cross-linked sodium alginate hydrogel, ROS: reactive oxygen species [83].

Drug treatment is the primary method for treating RA. Although significant progress has been made in relieving symptoms through drug treatment, there are still many drawbacks, such as difficulties in achieving sustained release, targeted delivery, and on-demand administration. Hydrogels, due to their excellent properties such as adjustability, biocompatibility, structural specificity, and stimuli-responsiveness, have gradually become drug carriers for delivering drugs to treat RA. Drug delivery hydrogels are mainly used to achieve sustained, continuous, and targeted release of drugs for RA to the inflamed joint sites [80,81]. For example, Xu et al. designed an injectable hydrogel combining a PEG matrix, hyaluronic acid, MPDANP, and MTX based on the fact that the antirheumatic drug MTX cannot be administered repeatedly. This hydrogel not only prolongs the release time and rate of MTX (demonstrating a continuous release period extending up to one month) but also regulates the self-release of ROS. Moreover, after a single intra-articular injection in the diseased joints of rats, the MPDANPs/MTX HA-PEG hydrogel exhibited significant anti-inflammatory effects, providing a certain reference for the clinical treatment of RA [82]. Wang et al. designed a double-dynamically crosslinked sodium alginate hydrogel (SPT@TPL) to overcome the extremely toxic nature of the antirheumatic drug triptolide (TPL) and the requirement for its long term administration. Gluconolactone is added to the mixed solution of the precursor prepared from sodium alginate and 3-aminophenylboronic acid for secondary cross-linking to form the SPT hydrogel. Then, triptolide is added to prepare the SPT@TPL. The dual dynamic crosslinked network enabled the hydrogel to quickly recombine with the surrounding groups and heal into a new network after being destroyed by external stimuli. Upon injection into the cartilage defect, this hydrogel can withstand and recover from the structural damage caused by frequent joint activities, which will prolong the hydrogel retention in the defect and extend the release time of triptolide, enhancing the potential for effective and durable treatment of RA. Figure 6 is a schematic diagram of the synthesis and functions of the SPT@TPL hydrogel for the treatment of RA. Based on the tight double-crosslinked structure of this hydrogel and the self-healing property resulting from the hydrogen bonds formed after the breakage of borate esters, TPL can maintain stability in the high ROS environment of RA and achieve a continuous release for up to 30 days. On day-30, the release concentration of TPL in the solution accumulated to 9.17 μg/mL. Through the dual effects of regulating ROS and the sustained release of TPL, SPT@TPL alleviates oxidative stress, promotes the reprogramming of macrophages to the M2 phenotype, and shows significant anti-inflammatory and joint cartilage regeneration effects in rat model experiments, providing a feasible option for the treatment of RA [83].

## 6. Hydrogels as Drug Carriers for the Treatment of RA via Different Administration Routes

A large number of studies have proven that hydrogels can be used as drug carriers for the treatment of RA. By designing the three-dimensional network structure of hydrogels, drugs can be released slowly, and the drug-loaded hydrogels can achieve precise and targeted release under specified conditions according to different stimulus signals such as pH, temperature, enzymes, light, and some other substances. For example, thermosensitive responsive hydrogels can change their size according to temperature variations. Pham et al. reported a biocompatible thermosensitive hybrid chitosan hydrogel containing gelatin and curcumin. The rheological study revealed that the coating agent (gelatin and curcumin) changed the hardness of the hydrogel from hard to soft. In addition, the hydrogel showed a unique gel-sol-gel transition at different temperatures when the gelatin content was 25 wt%. Furthermore, drug release experiments revealed that the hydrogel could sustain curcumin release (48 h, 30%) and had an excellent biocompatible scaffold. This avoids the disadvantages of direct drug administration, such as systemic toxicity, short drug efficacy, and frequent dosing [23,84,85,86,87]. Currently, drugs for the treatment of RA can be delivered through different administration routes, such as oral administration, intravenous injection, transdermal, ocular, rectal, subcutaneous injection, vaginal, and intra-articular injection. The most common administration routes for hydrogel drug carriers used in the treatment of RA are oral administration, parenteral administration (including intravenous injection and subcutaneous injection), transdermal, and intra-articular injection. Table 2 has summarized the advantages and disadvantages of the administration methods of drug-loaded hydrogels.

### 6.1. Oral Administration

Oral administration is a common method of drug administration. It refers to the route of administration in which drugs are taken through the mouth, absorbed by the gastrointestinal tract, and enter the bloodstream, thereby exerting their therapeutic effects. Currently, the vast majority of drugs for treating RA are administered orally. However, this can lead to the hydrolysis and inactivation of some protein-based drugs by the digestive tract proteases, thus affecting the treatment efficacy. Hydrogels, on the other hand, can encapsulate drugs inside and deliver them to the small intestine through the oral route, avoiding enzymatic hydrolysis of the drugs [94,95]. For example, Koetting et al. designed hydrogel microparticles composed of poly(itaconic acid-co-N-vinyl-2-pyrrolidone) with pH-responsive properties and demonstrated that the hydrogels with a lower crosslinking density were helpful for the loading of rituximab (a drug for treating RA). In vitro, each milligram of the hydrogel could load and release approximately 24 μg of rituximab. Although no additional experiments were conducted to explore its biological activity and human absorption rate, this provided a new idea that drug-loaded hydrogels could be used to treat RA through the oral route [88]. Carrillo-Conde et al. designed two pH-responsive hydrogel microparticle systems to deliver anti-TNF-α antibodies. One was P(MAA-g-EG) hydrogel microparticles polymerized from methacrylic acid (MAA) and ethylene glycol (EG), and the other was P(MAA-co-NVP) hydrogel microparticles copolymerized from MAA and N-vinylpyrrolidone (NVP). It was observed that the P(MAA-g-EG) hydrogel had a higher swelling ratio, which was beneficial for the delivery of large molecular weight proteins such as immunoglobulins. In addition, by using Sprague-Dawley (SD) rats as experimental subjects, the study also confirmed that these hydrogel microparticles could still release the anti-TNF-α antibodies into the small intestine under the low pH conditions of the gastrointestinal tract, protecting the antibodies from the harsh acidic environment of the gastrointestinal tract and releasing them in the neutral pH of the small intestine. The released antibodies still maintained their biological activity and were transported into the systemic circulation in the small intestine, which was conducive to the anti-TNF-α antibodies exerting their functions [89].

Although drug-loaded hydrogels can prevent protein-based drugs from being decomposed by pepsin and other substances when treating RA through the oral route and avoid the systemic toxicity caused by direct oral administration of drugs, there are still problems such as the difficulty in precisely controlling drug release and the possibility of causing certain irritation to the gastrointestinal mucosa. Moreover, the related technologies are not yet mature, and specific studies lack clinical trials for verification. Further research on the oral hydrogel systems for RA treatment is still needed to ensure their safety and high efficiency in treating RA.

### 6.2. Parenteral Administration

**Figure 7:**
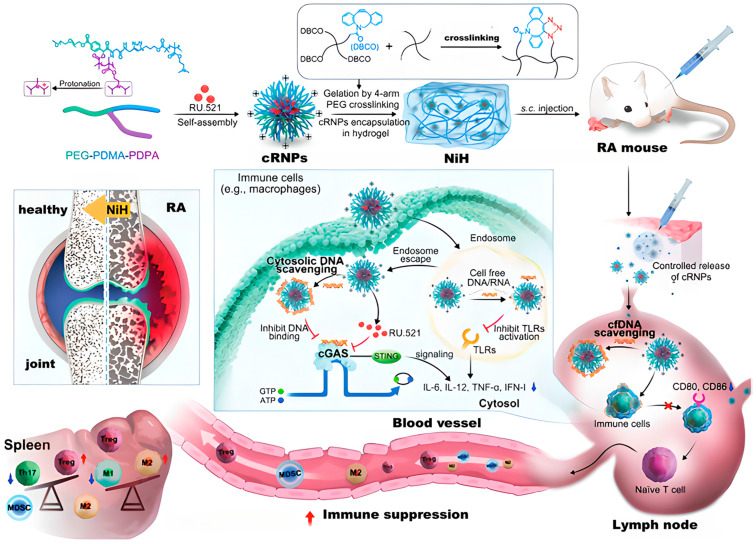
Design concept and treatment strategies of NiH, cfDNA: cell-free DNA, cGAS: cyclic guanosine monophosphate-adenosine monophosphate synthase, NiH: nanomedicine-in-hydrogel, CNP: cationic nanoparticles, CRNP: cNPs loaded with the potent cGAS inhibitor RU [91].

Parenteral administration involves injecting drugs for treating RA under the skin via needle injection, which avoids the influence of gastrointestinal factors and can quickly exert the drug’s efficacy and delay the drug release time [90]. For example, Cheng et al. designed a nanomedicine-in-hydrogel composite (NiH) that can simultaneously deliver cationic nanoparticles (cNPs) for scavenging cell-free DNA (cfDNA) and a cyclic guanosine monophosphate-adenosine monophosphate synthase (cGAS) inhibitor in order to scavenge cfDNA that can enhance the inflammatory response in RA and inhibit the related cGAS [91]. Figure 7 shows the design scheme of the hydrogel. They also confirmed through mouse experiments that there is a certain relationship between RA and lymphadenomegaly. Moreover, the selected cationic nanoparticles loaded with the potent cGAS inhibitor RU.521 (RU) were further fabricated into an injectable hydrogel. When subcutaneously injected into mice in the CIA mouse model, NiH delayed the deterioration of RA and reduced the severity of arthritis. Moreover, NiH can control drug release and enable the drug to remain in immunomodulatory tissues and cells for a long time, which is beneficial for the long term treatment of RA [91].

Intravenous injection is another way of parenteral administration for drug-loaded hydrogels. Feng et al. synthesized a methoxy poly(ethylene glycol)–poly(L-phenylalanine-co-L-cystine) (mPEG–P(LP-co-LC)) nanogel crosslinked by disulfide bonds to load the antirheumatic drug MTX. Since RA can lead to an oxygen-deficient joint microenvironment with a high concentration of the antioxidant glutathione (GSH), this hydrogel was designed as a redox-responsive system triggered by glutathione to release the drug. More methotrexate will be released in the presence of glutathione. In addition, NG/MTX has a selective biodistribution in the inflammatory joints of the CIA mouse model, which reduces the release of pro-inflammatory cytokines and effectively alleviates the progression of CIA, providing new insights for the future clinical application of stimuli-responsive hydrogels [96]. This hydrogel demonstrated long term stability, with no significant change in its mass. After being loaded into this drug delivery system, methotrexate showed the optimal duration of therapeutic action and the maximum therapeutic effect [96].

Although parenteral administration can release drugs under relatively stable conditions according to its design mechanism and enable the synergistic treatment of multiple drugs, there are still some problems, such as high preparation costs, slow onset of drug efficacy, possible adverse reactions caused by long term retention in subcutaneous tissues, and limited drug loading capacity requiring repeated injections. Therefore, improved solutions are needed to overcome these problems.

### 6.3. Transdermal Administration

**Figure 8:**
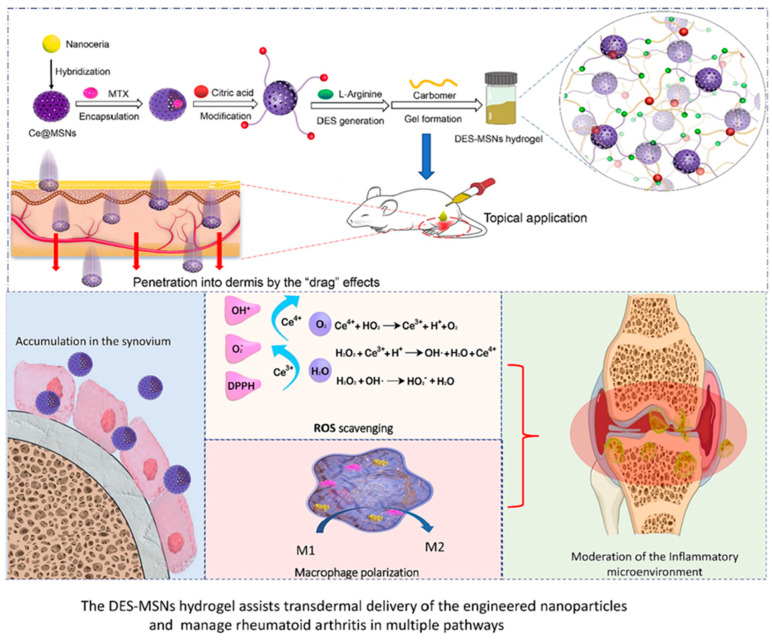
Schematic of transdermal delivery of engineered MSNs via DES hydrogel and the synergistic immune-chemotherapy effects on RA. The MSNs were decorated with nanoceria and encapsulated with MTX. Then, the nanoparticles were surface-modified with the HBD and co-heated with the HBA to generate the DES–MSNs. The system was engineered into hydrogel by reaction with carbomer 940. The DES hydrogel can “drag” the functionalized MSNs into deeper dermal layers and accumulate them at the RA sites. Consequently, a synergistic immune-chemotherapy of RA can be achieved by combining the ROS-scavenging and macrophage-reeducation properties of the functionalized NPs with the pharmacological capability of MTX [100]. Copyright © 2023 Elsevier B.V.

Transdermal administration serves as a convenient drug delivery approach for drug-loaded hydrogels in the treatment of RA. It eliminates the need for complicated procedures such as injection, allowing for self-controlled drug delivery. With its simplicity in operation and non-invasive nature, it has been increasingly adopted in the research on RA treatment [97,98]. For instance, Hou et al. incorporated the drug rutin (Rut) into black phosphorus nanosheets (BP) and subsequently integrated them into a matrix composed of hyaluronic acid and PVA to create a unique and innovative composite hydrogel named BP-Rut@Gel. Through relevant experiments, BP-Rut@Gel demonstrated excellent photothermal stability and also enhanced the penetration of the drug through the skin. In the in vitro drug release experiment, it was observed that continuous drug release occurred under slightly acidic conditions. Furthermore, in the experiment with the complete Freund’s adjuvant (CFA)-induced RA rat model, the levels of inflammatory factors in the serum samples were significantly decreased, verifying that the composite hydrogel can remarkably suppress RA-related inflammatory factors and possess high biocompatibility. The combination of the developed composite hydrogel with photothermal therapy offers novel perspectives for the research on RA treatment [99]. Likewise, Li et al. immobilized a nanoceria within a silica nanoparticle matrix (MSN) and encapsulated it with MTX. The functionalized nanoparticles were first engineered in an arginine-citric acid deep eutectic solvents (DESs) and then transferred to the carbomer hydrogel matrix. Eventually, they transferred it to a carbomer hydrogel matrix to obtain the DES-MSNs hydrogel. Figure 8 shows the design concept and treatment strategies of the DES-MSNs hydrogel system. Thanks to the strong interaction between the DES hydrogel and the lipids on the cell surface, the rigid MSN can penetrate through the skin layers without impairing the integrity of the dermis, thus facilitating the continuous penetration and accumulation of MSN at the sites affected by RA. The MTX carried by MSN can directly act on the affected areas, and nanoceria can initiate the scavenging of ROS and the transformation of macrophage phenotypes to modulate the inflammatory microenvironment, thereby achieving the synergistic treatment of drugs and immunomodulation. This provides a new strategy for non-invasive treatment of RA [100].

Although transdermal administration boasts advantages such as controllable drug delivery, simple operation, non-invasive treatment, and targeted therapy, it still suffers from several issues. These include limitations on drug absorption due to the skin barrier, limited drug loading capacity, an inability to control the release rate, which leads to slow drug efficacy, and potential skin adverse reactions caused by long term application. Consequently, further research is required to devise new solutions to address these problems.

### 6.4. Intra-Articular Administration

**Figure 9:**
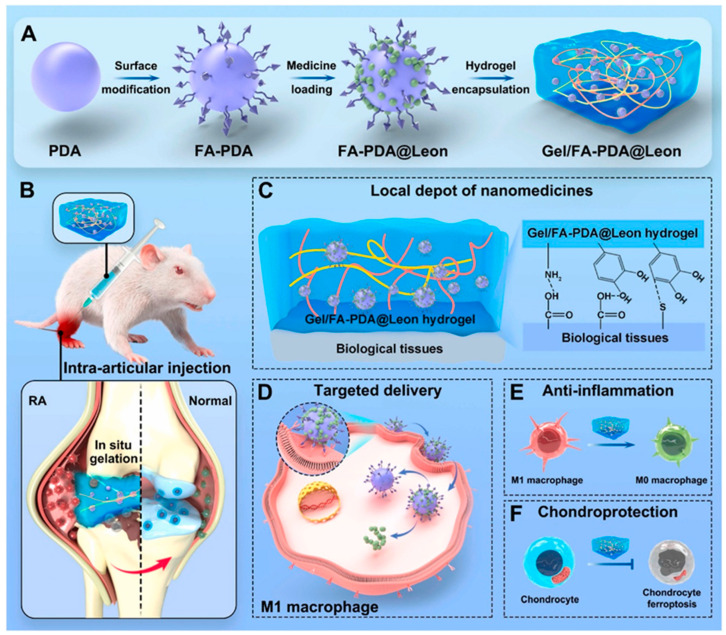
Schematic diagram of the medicine-loaded nanoparticle-encapsulated hydrogel for RA treatment. (**A**) Illustration of the preparation of the FA-PDA@Leon nanoparticles and the Gel/FA-PDA@Leon hydrogel. (**B**) The Gel/FA-PDA@Leon hydrogel is injected intra-articularly into the ankle joint, which is then in situ gelled in the cavity. (**C**) The in situ formed hydrogel is tightly bonded with surrounding tissues and serves as a depot of nanoparticles for prolonging their retention. (**D**) The released nanoparticles can target and enter into the M1 macrophages for targeted delivery of Leon. (**E**) The hydrogel reverses the M1 polarization of macrophage to suppress inflammatory response. (**F**) The hydrogel protects chondrocytes from ferroptosis [102]. Copyright © 2023 Elsevier B.V.

Intra-articular administration is a local treatment for RA, where drugs are directly injected into the affected joint cavities to avoid severe side effects from systemic drug circulation. Many studies have already verified its remarkable efficacy in treating RA [101]. For instance, Yang et al. proposed an injectable and bioadhesive hydrogel-mediated nanoparticle encapsulation strategy. They loaded Leonurine (Leon) onto folate-functionalized polydopamine (FA-PDA) nanoparticles to create FA-PDA@Leon nanoparticles, which were then encapsulated into an injectable and adhesive gelatin-based hydrogel matrix to form the anti-inflammatory and antioxidant Gel/FA-PDA@Leon hydrogel. The use of polydopamine (PDA)-based nanoparticles has several advantages. Firstly, PDA-based nanoparticles possess strong interfacial adhesion, allowing the immobilization of hydrophobic drugs through non-covalent bonds such as π-π stacking. Secondly, PDA-based nanoparticles can be easily modified with functional ligands or antibodies to enhance their ability to target and accumulate in specific tissues/cells, thus promoting the therapeutic effect of the loaded drugs. Thirdly, the antioxidant catechol groups on PDA can protect the drugs from reactive oxygen species damage and maintain their in vivo activity. Figure 9 is a schematic diagram of the drug-loaded nanoparticle-embedded hydrogel for the treatment of RA. Once injected into the diseased joints of mice, the hydrogel formed in situ and tightly bound to the surrounding tissues, preventing the nanoparticles from diffusing rapidly and prolonging the retention and treatment time. As the hydrogel gradually hydrolyzed, the folate modification enabled the nanoparticles to enter M1 macrophages, allowing Leon and the Gel/FA-PDA hydrogel to work together to inhibit inflammation-related pathways and reverse the M1 polarization of macrophages, thus reducing the secretion of related inflammatory factors. This injectable hydrogel not only extends the drug treatment time but also helps protect chondrocytes and restore joint functions [102]. Xu et al. developed a biomacromolecular hydrogel with apoferritin nanoparticles, namely oxidized chondroitin sulfate-chitosan-sodium glycerol β-phosphate-fibronectin/small interfering high mobility group protein (OCF/siHMGB1). Given that the imbalance between pro-inflammatory M1 and anti-inflammatory M2 macrophages can worsen RA, while the polarization of M1 macrophages to the M2 phenotype can relieve its symptoms, this polarization is a potential treatment approach for RA. After injecting the hydrogel containing Fn/siHMGB1 nanoparticles into the diseased joints of mice, the hydrogel quickly solidified, and the Fn/siHMGB1 nanoparticles were continuously released into the joint tissues. Once taken up by M1 macrophages, siHMGB1 in the nanoparticles induced the macrophages to polarize from the pro-inflammatory M1 phenotype to the anti-inflammatory M2 phenotype, thereby producing a significant anti-inflammatory effect for RA treatment [103].

Although intra-articular administration enables precise drug delivery, has a quick drug effect, and avoids systemic toxicity from direct oral administration, its operation is complex and demands high technical skills. Moreover, multiple injections are needed, and there is an infection risk, which restricts its application in RA treatment. Hence, relevant innovations and research are required to ensure its safety and high efficiency in treating RA.

### 6.5. Application Prospects and Challenges

#### 6.5.1. Scalability Challenge

Expand the production scale difficulty: The synthesis and preparation of hydrogels are often easier to achieve in small-scale laboratory conditions, but to expand to large-scale production, there will be problems such as process parameter optimization and quality control. For example, the conditions such as temperature and mixing speeds that can be precisely controlled in the laboratory are difficult to achieve uniformly in large-scale production reactors, which may lead to differences in the performance of hydrogels between batches.

Limited scale of clinical application: there is a large group of arthritis patients. To meet the large-scale demand for clinical treatment, hydrogel materials need to have good scalability in production technology. However, at present, some advanced hydrogel preparation technologies, such as personalized hydrogel stent manufacturing based on 3D printing, are difficult to rapidly expand production to meet the needs of a large number of patients, although they have significant effects on personalized treatment.

#### 6.5.2. Manufacturing Cost Challenge

High cost of raw materials: many hydrogel materials used to treat arthritis need to use special biocompatible polymers, growth factors, or active pharmaceutical ingredients, which are often expensive. For example, some natural polymers such as chitosan and hyaluronic acid have complex extraction and purification processes, resulting in high costs; however, synthetic polymer materials such as polyethylene glycol have relatively high costs to achieve medical grade purity and quality standards.

Complex preparation process: The preparation process of hydrogel may involve complex chemical reactions, cross-linking processes, or special processing technology, which increases the manufacturing cost. Taking the photo crosslinked hydrogel as an example, light sources and photoinitiators with specific wavelengths are required. The equipment investment and operation costs are high, and the residual photoinitiators may have an impact on the product quality, which requires additional purification steps, further increasing the cost.

#### 6.5.3. Long Term Internal Stability Challenge

Influence of physiological environment: the physiological environment of human joints is complex, including factors such as enzymes, electrolytes, and pH in joint fluid, which may affect the stability of hydrogel. For example, the protease in the joint fluid may degrade the protein components in the hydrogel, leading to structural damage and performance degradation of the hydrogel; the change of electrolyte concentration may affect the swelling behavior of hydrogels, making them unable to maintain stable morphology and function.

Changes in mechanical properties: the movement and force of the joints of arthritis patients are complex, and the hydrogel needs to remain stable under the long term pressure, friction, and other mechanical forces generated by joint movement. However, in practical applications, hydrogels may suffer from fatigue, fracture, or wear due to repeated mechanical action. For example, the hydrogel scaffold used to repair articular cartilage may gradually deform and damage during joint movement, affecting the treatment effect.

#### 6.5.4. Potential Immunogenicity Challenge

Immunogenicity of materials: although most hydrogel materials are designed to have good biocompatibility, some materials may cause an immune reaction. For example, the chemical structure of some synthetic polymer hydrogels may be recognized by the immune system as foreign antigens, thus activating immune cells and triggering inflammatory reactions. Even natural polymer hydrogels, their sources, and impurities in the preparation process may also lead to the increase of immunogenicity.

Effects of degradation products: hydrogels will gradually degrade in vivo, and their degradation products may have potential immunogenicity. If the degradation products cannot be metabolized or excreted by the human body in a timely manner, they may accumulate in local tissues, stimulating the immune system to produce an immune response. For example, some small molecular fragments generated by the degradation of hydrogels may activate immune cells such as macrophages, release inflammatory factors, and cause local tissue inflammation and immune damage.

## 7. Conclusions and Future Prospects

The functions and administration modalities of hydrogels in the treatment of RA were discussed in this article, and the frontier research findings regarding hydrogels’ application in this field were reviewed. It not only comprehensively expounds on the outstanding characteristics of hydrogels but also critically reviews the practical implementations of hydrogels with diverse functions in the treatment of RA. In addition, the utilization of hydrogels as drug carriers has also been deeply explored in the treatment of RA through various drug delivery routes. Although remarkable progress has been achieved in the treatment of RA with hydrogels, such as the enhancement of drug loading efficiency and the circumvention of systemic toxicity caused by oral medications, there are still several formidable challenges. These encompass the incapability of fabricating multifunctional hydrogels, the potential induction of autoimmune responses by hydrogels within the body, the lack of assurance regarding the in vivo stability of hydrogels, and the side effects that long term usage may lead to systemic toxicity. Obviously, these challenges impose substantial constraints on the development prospects of hydrogels in the treatment of RA. At present, the application of hydrogels in RA treatment is still in an embryonic stage of development, and there are still many problems to be resolved, especially in the treatment effect and the treatment scheme design. It is expected that in the next few years, the research endeavors on hydrogels for RA treatment will increase significantly. Novel hydrogel polymers are expected to be explored, and multifunctional hydrogels that can simultaneously play the functions of immune regulation, drug release, and tissue repair will be designed. Moreover, artificial intelligence technology will be ingeniously incorporated into the hydrogel design for the treatment of RA. In addition, new advanced materials will be developed and synergistically combined with hydrogels to offset the existing deficiencies of hydrogels so that hydrogels can effectively treat RA and help patients get rid of this affliction.

## Figures and Tables

**Table 1:** Functions of hydrogels used for the treatment of RA.

Type	Function	Matrix Material	Ref.
Tissue engineering scaffold hydrogels	It is used to provide support for the repair of tissues such as articular cartilage and synovium. The three-dimensional structure of hydrogels can mimic the environment of the extracellular matrix, which is beneficial to cell adhesion, proliferation, and differentiation.	Natural materials such as polymers such as chitosan, alginate, and hyaluronic acid, proteins (gelatin, collagen, silk fibroin), as well as polymers such as PVA and PEG.	[68]
Lubricating hydrogels	It can mimic the lubricating properties of natural synovial fluid and act as “artificial synovial fluid” in the joint cavity. The water phase and the hydrophilic polymer network inside it endow it with good lubricating ability, which can reduce the friction coefficient during joint activities and minimize wear and tear.	Natural materials such as polymers such as chitosan, alginate, and hyaluronic acid, proteins (gelatin, collagen, silk fibroin), as well as polymers such as PVA and PEG.	[69]
Immunomodulatory hydrogels	Regulate the local immune microenvironment of joints by means of adsorbing or neutralizing inflammatory cytokines and interfering with the activation and proliferation of immune cells.	Natural materials such as polymers such as chitosan, alginate, and hyaluronic acid, proteins (gelatin, collagen, silk fibroin), as well as polymers such as PVA and PEG.	[70]
Drug delivery hydrogels	Achieve the slow and controllable release of drugs, and utilize its own unique network structure or physicochemical properties to encapsulate the drugs for treating RA.	Natural materials such as polymers such as chitosan, alginate, hyaluronic acid, proteins (gelatin, collagen, silk fibroin), as well as polymers such as PVA and PEG.	[71]

**Table 2:** Advantages and Disadvantages of Different Administration Routes of Drug-Loaded Hydrogels for the Treatment of RA.

Type	Advantage	Disadvantage	Ref.
Oral administration	It is convenient and easy to use. Patients can take it on their own without the need for any invasive procedures. With the drugs encapsulated in hydrogels, their stability is enhanced, and they can exert their effects mainly in the local area of the intestine, thus avoiding the systemic toxicity that may be caused by direct oral administration.	It is rather difficult to control the precision of drug release, and it may also cause certain irritation to the gastrointestinal mucosa.	[88,89]
SC injection	It is easy to operate with low invasiveness. The drugs can be slowly released under the skin, and the drug release rate is controllable, which is beneficial for maintaining a stable drug efficacy.	The drugs take effect relatively slowly. Affected by individual differences in subcutaneous tissues, remaining in the subcutaneous tissues for a long time may cause some adverse reactions. Moreover, the drug loading capacity is limited, and repeated injections are required.	[90]
IV injection	It takes effect quickly and enables targeted delivery, realizes systemic immune regulation, and improves the condition of RA as a whole.	It has relatively high risks, strict requirements for hydrogels, and relatively high preparation costs.	[91]
Transdermal	It is non-invasive and convenient. The drugs can act directly on the local tissues around the joints, precisely target joint inflammation, reduce systemic side effects, and the drug delivery can be controlled at any time.	The skin barrier restricts drug absorption. The drug release rate is affected by the physiological state of the skin. Long term application may cause adverse skin reactions.	[92]
Intra-articular injection	Drugs can act precisely within the affected joints, release drugs in a long acting and slow manner, and reduce systemic side effects.	It is difficult to operate, there is a risk of infection, and individual differences affect drug release.	[93]

## Data Availability

Data will be made available on request.

## Abbreviations

The following abbreviations are used in this manuscript:

RA	rheumatoid arthritis
NSAIDs	non-steroidal anti-inflammatory drugs
DMARDs	disease-modifying antirheumatic drugs
SNP	single nucleotide polymorphisms
HLP	Human leukocyte antigen
CTL	cytotoxic T lymphocytes
APC	Antigen-presenting cells
RF	rheumatoid factor
ACPA	anti-citrullinated protein antibodies
TNF-α	tumor necrosis factor-α
IL-1	interleukin-1
IL-6	interleukin-6
MMPs	matrix metalloproteinases
MTX	methotrexate
PVA	polyvinyl alcohol
PEG	polyethylene glycol
H_2_O_2_	hydrogen peroxide
PVA/PEG/GO	polyvinyl alcohol/polyethylene glycol/graphene oxide
ROS	reactive oxygen species
CM	metformin-loaded hollow copper sulfide
NPs	nanoparticles
siBiMPNH	pH-responsive injectable peptide hydrogel
IL-1β	interleukin-1β
MPDANP	mesoporous polydopamine nanoparticles
SPT@TPL	double-dynamically crosslinked sodium alginate hydrogel
TPL	antirheumatic drug triptolide
MAA	methacrylic acid
EG	ethylene glycol
NVP	N-vinylpyrrolidone
NiH	nanomedicine-in-hydrogel composite
cNPs	cationic nanoparticles
cfDNA	cell-free DNA
cGAS	cyclic guanosine monophosphate-adenosine monophosphate synthase
CIA	collagen-induced arthritis
mPEG–P(LP-co-LC)	methoxy poly(ethylene glycol)–poly(L-phenylalanine-co-L-cystine)
MSN	silica nanoparticle matrix
DES	deep eutectic solvents
Leon	Leonurine
FA-PDA	folate-functionalized polydopamine
OCF/siHMGB1	oxidized chondroitin sulfate-chitosan-sodium glycerol β-phosphate-fibronectin/small interfering high mobility group protein
